# circRNA_17725 Promotes Macrophage Polarization towards M2 by Targeting FAM46C to Alleviate Arthritis

**DOI:** 10.1155/2023/6818524

**Published:** 2023-03-31

**Authors:** Chunjuan Yang, Biao Ni, Chaoran Li, Wenchang Sun, Zhangxue Wang, Hui Wang, Xinyue Hou, Shushan Yan, Xiaodong Wang, Donghua Xu

**Affiliations:** ^1^Department of Rheumatology of the First Affiliated Hospital & the First Clinical College, Weifang Medical University, Weifang 261000, China; ^2^Central Laboratory of the First Affiliated Hospital & the First Clinical College, Weifang Medical University, Weifang 261000, China; ^3^Department of Gastrointestinal and Anal Diseases Surgery of the Affiliated Hospital, Weifang Medical University, Weifang 261000, China; ^4^Department of Rheumatology of the Affiliated Hospital, Weifang Medical University, Weifang 261000, China

## Abstract

Accumulating studies have implicated that circular RNAs (circRNAs) play vital roles in the pathogenesis of rheumatoid arthritis (RA). Dysregulation of macrophage polarization leads to immune homeostatic imbalance in RA. However, the altering effects and mechanisms of circRNAs on macrophages polarization and immune homeostatic balance remain largely unclear. We aimed to investigate the potential role of circRNA_17725 in RA. The high-throughput sequence was performed to identify the dysregulated circRNAs in RA. We confirmed the data by CCK-8, EdU, and Annexin V/PI staining to elucidate the proliferation and apoptosis. The expressions of M1/M2-associated markers were confirmed using real-time PCR and flow cytometry analysis. Luciferase reporter assay and RNA Binding Protein Immunoprecipitation (RIP) were used to demonstrate the underlying mechanism of circRNA_17725. The altering effect of circRNA_17725 on macrophages in vivo was evaluated using collagen-induced arthritis (CIA) mouse model. circRNA_17725 was demonstrated to be downregulated in peripheral blood mononuclear cells and CD14^+^ monocytes from RA cases in contrast to healthy controls. The negative association between circRNA_17725 and the disease activity indexes (CRP, ESR, and DAS28) was observed, suggesting a vital role of circRNA_17725 in RA disease activity. Besides, after a coexpression analysis based on high-input sequencing and the bioinformatics analysis in MiRanda and TargetScan databases, a circRNA_17725-miR-4668-5p-FAM46C competing endogenous RNA (ceRNA) network was hypothesized. A series of cytology experiments *in vitro* have implicated that circRNA_17725 could inhibit the proliferation but enhance the apoptosis of macrophages. Decreased expression of TNF-*α*, IL-1*β*, and MMP-9 were observed in the supernatant of circRNA_17725-overexpressed Raw264.7 macrophages, implicating the inhibitory effect of circRNA_17725 on macrophage inflammatory mediators. Furthermore, circRNA_17725 could promote macrophage polarization towards M2 by targeting miR-4668-5p/FAM46C as a miRNA sponge. Additionally, circRNA_17725-overexpressed macrophages alleviated arthritis and protected against joint injuries and bone destruction by inducing macrophage polarization towards M2 in collagen-induced arthritis (CIA) mice. This study has suggested that circRNA_17725 regulated macrophage proliferation, apoptosis, inflammation, and polarization by sponging miR-4668-5p and upregulating FAM46C in RA.

## 1. Introduction

Rheumatoid arthritis (RA) is a common and chronic autoimmune disease. It can cause synovial hyperplasia, inflammation, cartilage injury, bone damage, and even disability [1]. The pathogenesis of RA is complex with dysregulated mononuclear macrophages, T cells, and B cells. It has been well-documented imbalance of M1/M2 macrophage, Th17/Treg, and other immune cells leads to immune homeostatic imbalance. M1 macrophage is associated with enhanced inflammation and immune activation in specific tissues and organs in RA. It has been demonstrated that genetics, sex hormones, and infectious factors are closely related to RA [2]. Chronic inflammation affects the onset and progression of RA. In spite of great advance in RA diagnosis and treatment, illustrating the molecular mechanism underlying RA pathogenesis is essential for identifying more valuable therapeutic targets for RA. Exploring more effective immunotherapy based on immune cells and potential biological targets has always been the research focus in RA.

During the past few years, the role of noncoding RNAs (ncRNAs) in autoimmune diseases has been drawing more and more attention, primarily including circular RNA (circRNA), long chain noncoding RNAs (lncRNA), and microRNAs (miRNA) [3]. These ncRNAs play critical roles in maintaining the stability and normal expression of genes. Furthermore, ncRNAs regulate inflammation and autoimmunity through protein-RNA or RNA-RNA interactions. As a key type of ncRNA, circRNAs have been suggested to contribute to RA by a number of studies [4, 5]. Some studies have implicated that some circRNAs are specifically expressed in RA, such as circ_0008360, circ_0140271, and circ_0003972 [6–8], which are primarily involved in regulating inflammation and autoimmune disorders. circRNA is a new competitive endogenous RNA (ceRNA) with high stability. They are critical regulators in the immune system, and are specifically expressed in specific organs, tissues, or cells. Accumulating evidence has highlighted the crucial potentials of circRNAs as diagnostic and therapeutic markers in multiple autoimmune diseases, including systemic lupus erythematosus (SLE), multiple sclerosis (MS), and RA [9–11]. Nevertheless, little is known of the effects and mechanism of circRNAs in regulating immune cell polarization and immune homeostatic imbalance. As described previously [12], we have demonstrated several circRNAs are aberrantly expressed in RA, including circRNA_09505 and circRNA_17725. circRNA_09505 has been suggested to enhance inflammation injuries and bone damages in collagen-induced arthritis (CIA) mice by sponging miR-6089 as a ceRNA through the AKT1/NF-*κ*B signaling pathway [12]. circRNA_17725 was significantly downregulated in RA with unclear regulatory effects and molecular mechanisms. Here, we focus on the altering effects of circRNA_17725 in RA pathogenesis and progression, particularly highlighting its potential use in RA as a key biomarker.

## 2. Materials and Methods

### 2.1. Sample Preparation and Cell Culture

The study regarding human subjects had been approved by the Institutional Ethics Committee of the First Affiliated Hospital, Weifang Medical University (2021YX076). Peripheral blood mononuclear cells (PBMCs) were isolated by use of Ficoll-Paque gradient centrifugation from 35 RA cases and 28 healthy controls adjusted by age and sex. They had all agreed with the written informed consent. Raw264.7 cells were incubated in DMEM adding with 10% fetal bovine serum (Sigma-Aldrich, USA) under 37°C and 5% CO_2_. Cells were transfected by miRNA mimics and mimics controls (Ruibo Biosciences, Guangzhou, China). Lentivirus plasmids overexpressing circRNA_17725 and plasmids of FAM46C-WT (wild-type) and FAM46C-MT (mutant) were constructed by OBiO Technology Corp., Ltd. (Shanghai, China). Raw264.7 macrophages were transfected by these plasmids and used for experiments. The luciferase activity in macrophages was estimated by the luminescence kit (Toyo Ink, Japan) according to the protocol as reported previously [12].

### 2.2. High-Throughput Sequencing Analysis

As described previously, we performed the high-throughput sequence to identify the dysregulated circRNAs in RA based on the platform of Oebiotech Company (Shanghai, China) [12]. The correlation coefficient was analyzed according to the complementary base pairs of circRNA_17725/mRNAs with shared miRNAs and the coexpression of circRNA_17725/mRNAs/miRNAs in RA PBMCs. circRNA was determined to have a positive coexpression correlation with mRNA, when the *P* value was less than 0.05 and the absolute value of Pearson's correlation coefficient was more than 0.7. Pairs of circRNAs-miRNAs-mRNAs were screened according to the principles of sharing miRNAs ≥ 3, Pearson's correlation index ≥ 0.7, and *P* < 0.05.

### 2.3. Cell Proliferation and Apoptosis

Briefly, Raw264.7 macrophages (2 × 10^5^/well) were seeded into cell plate in fetal bovine serum-free DMEM overnight. Subsequently, the proliferation of macrophages at 24, 48, and 72 hours was estimated using CCK-8 kit (Vazyme Biotech, Nanjing, China). Besides, we used the 5-ethynyl-2′-deoxyuridine (EdU) kit to evaluate the proliferation of cells at 48 h according to the protocol of the reagent kit (RiboBio, Guangzhou, China). In addition, cells were pretreated with H_2_O_2_ (100 mmol/L) for 4 hours. Flow cytometry was carried out to estimate the impact of circRNA_17725 on the apoptosis of macrophage.

### 2.4. Real-Time Polymerase Chain Reaction (PCR)

PBMCs from another 40 RA cases and 30 controls enrolled in hospital for health examination were purified by CD14^+^ magnetic bead sorting (Miltenyi Biotec, San Diego, USA) after isolating by the Ficoll-Paque gradient centrifugation. The expression of circRNA_17725 in CD14^+^ monocytes was also determined by real-time PCR. The association between circRNA_17725 and IL-10 and FAM46C and TNF-*α* was estimated. We applied TRIzol (Invitrogen, CA, USA) to isolate total RNAs, which were used for the synthesis of cDNA as template (0.5 *μ*g) by use of RT kit (Vazyme, Nanjing China). cDNAs (5 ng) were used as templates for PCR using SYBR Green Mastermix kit (Vazyme, Nanjing China). The Novabio SYBR qPCR Mix kit (Novabio, Shanghai, China) was used to amplify circRNA. TaqMan miRNA PCR kit (ThermoFisher Scientific, USA) was applied for miRNA determination. Primers were as follows: circRNA_17725-f, AGGGAGAAAGCTTGATATGAGTTTG3, circRNA_17725-r, AGAAGTAATAAAGCCAGCAGGTACG3; human CD206-f, GGGTTGCTATCACTCTCTATGC, human CD206-r, TTTCTTGTCTGTTGCCGTAGTT; human IL-10-f, GACTTTAAGGGTTACCTGGGTTG, human IL-10-r, TCACATGCGCCTTGATGTCTG; human TNF-*α*-f, CCTCTCTCTAATCAGCCCTCTG, human TNF-*α*-r, GAGGACCTGGGAGTAGATGAG; human GAPDH-f, GGAGCGAGATCCCTCCAAAAT, human GAPDH-r, GGCTGTTGTCATACTTCTCATGG; human FAM46C-f, GGCCACGTTTTGGTCAAAGAC, human FAM46C-r, GGGAACACAGAACCACATCTC; mouse FAM46C-f, AACTGGGATCAGGTTAGCCG, mouse FAM46C-r, CAACCCAAGCCGTTGTCTT; mouse CD11c-f, CTGGATAGCCTTTCTTCTGCTG, mouse CD11c-r, GCACACTGTGTCCGAACTCA; mouse CD163-f,GGTGGACACAGAATGGTTCTTC, mouse CD163-r, CCAGGAGCGTTAGTGACAGC; and mouse GAPDH-f, AGGTCGGTGTGAACGGATTTG, mouse GAPDH-r, TGTAGACCATGTAGTTGAGGTCA.

### 2.5. Flow Cytometry Analysis

Flow cytometry was carried out to determine the genotypes in Raw264.7 cells and mice spleen cells. 2 × 10^5^ cells were resuspended in 100 *μ*L PBS incubating with 5 *μ*L/anti-mouse CD14, HLA-DR, CD68, CD206, CD11c, and CD163 fluorescent-labeled antibodies (BioLegend, USA) for 30 min at room temperature. After washing twice using 300 *μ*L PBS, cells were determined by flow cytometry. For apoptotic analysis, cells were stimulated by H_2_O_2_ (100 *μ*mol/L) for 4 h and harvested by centrifugation. Then, cells were resuspended in 1 × Binding Buffer plus apoptosis detection reagents (5 *μ*L/tube) according to the protocol of Annexin V/PI Apoptosis Assay kit (Multisciences Biotech., Hangzhou, China). After incubation for 15 min at room temperature, the apoptosis status of cells was estimated by flow cytometry.

### 2.6. Enzyme-Linked Immunosorbent Assay (ELISA)

2 × 10^5^/well Raw264.7 macrophage was seeded into 96-cell plate in serum-free DMEM at 37°C, 5% CO_2_. Cells were stimulated by LPS (1 *μ*g/mL) for 48 h. Cytokines in the cultural supernatant were detected by use of mouse TNF-*α*, IL-10, IL-1*β* (R&D Systems, USA), and MMP-9 (abcam, USA) ELISA kits according to the protocols, as decried previously [12]. In addition, TNF-*α* and IL-1*β* in the plasm samples from mice were also determined.

### 2.7. RNA Fluorescence In Situ Hybridization (FISH)

5 × 10^5^/well Raw264.7 cells were seeded into plastic dish (35 mm, Thermo Scientific Nunc, USA) overnight adding with DMEM without fetal bovine serum. Subsequently, Raw264.7 cells were fixed with paraformaldehyde (4%) and dehydrated using ethanol. We carried out FISH to estimate the location of circRNA_17725 in Raw264.7 cells, which were labeled by fluorescent probe for circRNA_17725 during the process of hybridization.

### 2.8. Luciferase Reporter Assay

5 × 10^5^/well 293T cells seeded into cell culture plate, which were incubated until the cell confluence up to 70-80%. Then, cells were transfected by miRNA mimics, or mimics controls, or luciferase reporter plasmids of FAM46C-WT and FAM46C-MT by use of the lipofectamine 2000. At last, 293T cells were lysed. We applied the Picagene Dual SeaPansy luminescence kit (Toyo Ink, Japan) to detect the luciferase activity in cells according to the protocol.

### 2.9. RNA Binding Protein Immunoprecipitation (RIP)

RIP was performed to estimate the association between circRNA_17725 and miR-4668-5p. Briefly, 5 ~ 10 × 10^6^ macrophages were lysed. Lysates were collected by centrifugation, followed by probe incubation based on the instructions of RIP Kit (Millipore, Bedford, USA). Ago2 and IgG were used for immunoprecipitation with cell lysates. Real-time PCR was conducted to determine the expression of circRNA_17725 and miR-4668-5p.

### 2.10. Collagen-Induced Arthritis (CIA) Mouse Model Construction and Estimation

Animal experiments were conducted according to the guidelines of Institutional Animal Care and Use Committee of Weifang Medical University (2021SDL311). We used bovine type II collagen (4 mg/mL, Chondrex, Washington, USA) and the Freund's complete adjuvant (Sigma-Aldrich, USA) to construct CIA mice model by tail vein injection at the ratio of 1 : 1. There were 6 mice in each group. Mice were intravenously injected with Raw264.7 cells (10^6^ cells in 200 *μ*L PBS) on day 5 and day 15. Booster immunity was conducted by use of 100 *μ*L emulsion solution on day 21. After estimating for 60 days from the first immunization, all mice were executed for determination. We carried out Hematoxylin-Eosin (H&E) and Safranin O/Solid green staining to estimate the status of mouse joints and tissues.

### 2.11. Statistical Analysis

All data was analyzed by SPSS (v20.0) software and GraphPad Prism (v8.0) software. The data were analyzed by parametric or nonparametric tests according the homoscedasticity estimation and the adherence to the normal curve. The parametric tests were performed using one-way ANOVA or independent sample Student's *T* test. The nonparametric tests were conducted using Mann–Whitney *U* test or Kruskal-Wallis *H* test according to subgroups. Pearson's correlation analysis estimated the correlation between circRNA_17725 expression and clinical indexes of CRP, ESR, and DAS28. *P* < 0.05 was statistically significant.

## 3. Results

### 3.1. Expression of circRNA_17725 in the Peripheral Blood Mononuclear Cells and Its Association with Clinical Indexes

**Figure 1:**
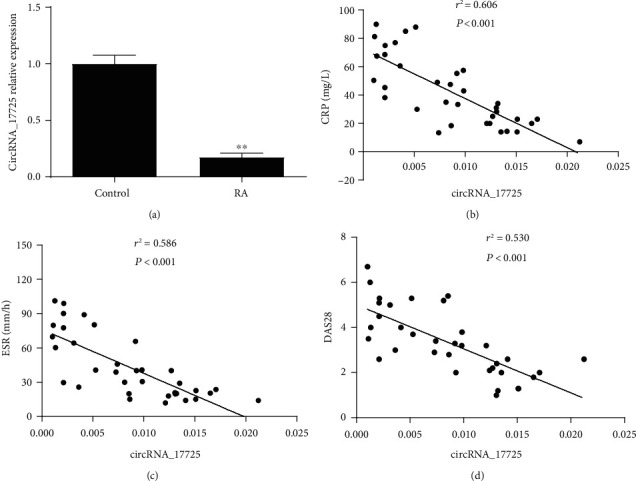
Expression of circRNA_17725 in the PBMCs and its association with the disease activity indexes of RA. (a) Expression of circRNA_17725 in the PBMCs from RA patients (^∗∗^*P* < 0.01; RA/control: 35/28). (b) Pearson's correlation analysis: relationship of circRNA_17725 with CRP (*n* = 35). (c) Pearson's correlation analysis: relationship of circRNA_17725 with ESR (*n* = 35). (d) Pearson's correlation analysis: relationship of circRNA_17725 with DAS28 (*n* = 35).

In a previous study, we found that circRNA_17725 was significantly reduced in RA as implicated by gene sequencing analysis and real-time PCR [12]. In this study, circRNA_17725 was further demonstrated to be significantly decreased in RA patients' PBMCs (Figure 1(a)). Besides, the expression of circRNA_17725 was negatively associated with the disease activity indexes including CRP, ESR and DAS28 (Figures 1(b)–1(d)). Taken together, circRNA_17725 may play a critical role in the pathogenesis or disease progression of RA.

### 3.2. Potential Target Prediction of circRNA_17725 in Macrophage

**Figure 2:**
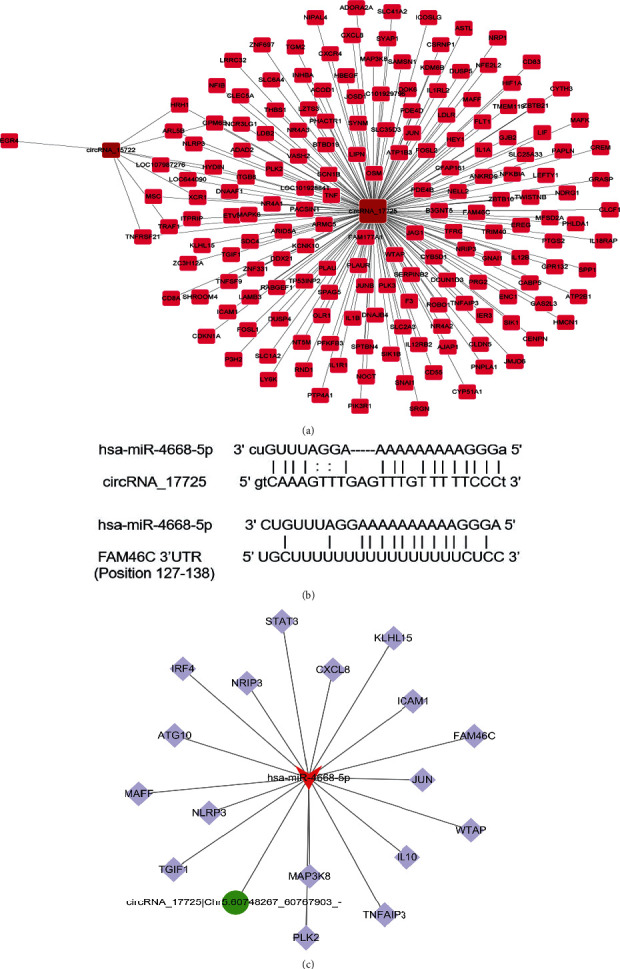
Potential targets prediction of circRNA_17725. (a) Positive coexpression association between circRNA_17725 and FAM46C and other factors implicated by high-throughput sequencing and bioinformatics analysis. (b) Complementary base pairs of circRNA_17725 and miR-4668-5p, plus miR-4668-5p, and FAM46C. (c) A predicted circRNA_17725/miR-4668-5p/FAM46C ceRNA network (the red shape represents miR-4668-5p, the purple squares represent targeted mRNAs including FAM46C, and the green circle represents circRNA_17725).

The bioinformatics analysis was conducted to evaluate the potential targets of circRNA_17725. Based on the expression value analysis of high-throughput sequencing, a positive coexpression relationship between circRNA_17725 and FAM46C was observed (Figure 2(a)), which was a macrophage polarization-associated molecule. However, whether circRNA_17725 directly binds to FAM46C needs further demonstration. Competing endogenous RNA (ceRNA) is the main mechanism for circRNA to exert biological effects. We subsequently found that circRNA_17725 and miR-4668-5p possessed 16 complementary base pairs with miR-4668-5p after scanning in MiRanda and TargetScan databases (Figure 2(b)). Moreover, miR-4668-5p was predicted to recognize the 3′ untranslated region (UTR) of FAM46C scanned in TargetScan database (Figure 2(b)). Accordingly, we hypothesize that circRNA_17725 may serve as a ceRNA molecule competitively antagonizing miR-4668-5p and targeting macrophage polarization-related molecule, namely, FAM46C (Figure 2(c)). Whether the circRNA_17725/miR-4668-5p/FAM46C contributes to macrophage polarization and immune balance in RA warrants to be explored in subsequent experiments.

### 3.3. Positive Association between circRNA_17725 and FAM46C

**Figure 3:**
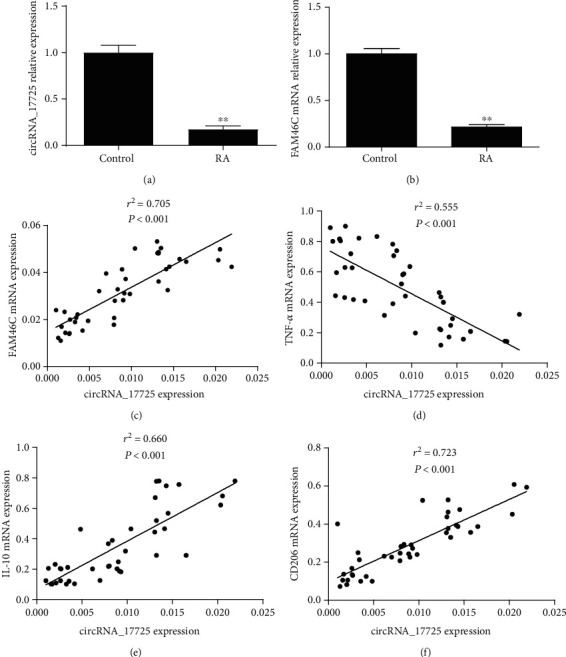
Association between circRNA_17725, FAM46C, TNF-*α*, IL-10, and CD206 in RA. (a) Expression of circRNA_17725 in CD14^+^monocytes from RA patients in contrast to controls (^∗∗^*P* < 0.01; RA/control: 40/30). (b) Expression of FAM46C in CD14^+^monocytes from RA patients in contrast to controls (^∗∗^*P* < 0.01; RA/control: 40/30). (c) Pearson's correlation analysis: relationship of circRNA_17725 with FAM46C (*n* = 40). (d) Pearson's correlation analysis: relationship of circRNA_17725 with TNF-*α* (*n* = 40). (e) Pearson's correlation analysis: relationship of circRNA_17725 with IL-10 (*n* = 40). (f) Pearson's correlation analysis: relationship of circRNA_17725 with CD206 (*n* = 40).

FAM46C is a M2 macrophage polarization-related molecule. In this study, mRNAs of circRNA_17725 and FAM46C were both significantly reduced in CD14^+^ monocytes of RA patients compared with those in controls (Figures 3(a) and 3(b)). FAM46C was positively associated with circRNA_17725 demonstrated by Pearson's correlation analysis (Figure 3(c)). Moreover, the expression of circRNA_17725 was positively related to IL-10 and CD206, but negatively associated with TNF-*α* regarding their expression at mRNA levels (Figures 3(d)–3(f)). As mentioned before, IL-10, CD206, and FAM46C are typical markers for M2 cells, while TNF-*α* is a M1 maker. M2 macrophage can inhibit inflammation and exert immunoregulatory effects in maintaining homeostasis. Accordingly, we hypothesize that circRNA_17725 may contribute to M2 polarization by targeting FAM46C.

### 3.4. circRNA_17725 Location and Its Regulatory Effects on Macrophage Proliferation, Apoptosis, and Inflammation

**Figure 4:**
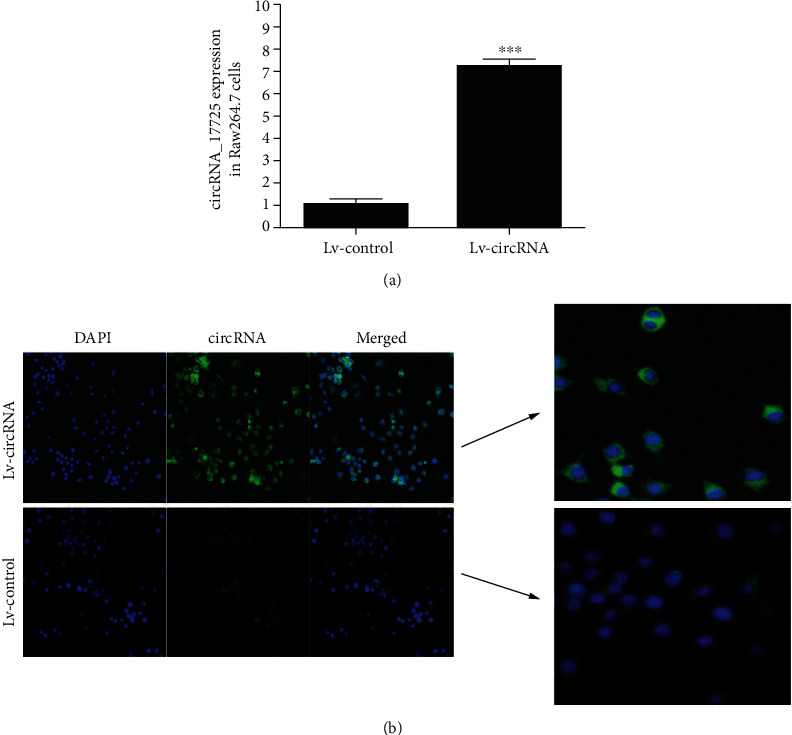
Location of circRNA_17725 in macrophages. (a) Real-time PCR: circRNA_17725 expression in Raw264.7 macrophages (^∗∗∗^*P* < 0.001; *n* = 3). (b) FISH: location of circRNA_17725 in Raw264.7 macrophages (representative pictures; 20x).

**Figure 5:**
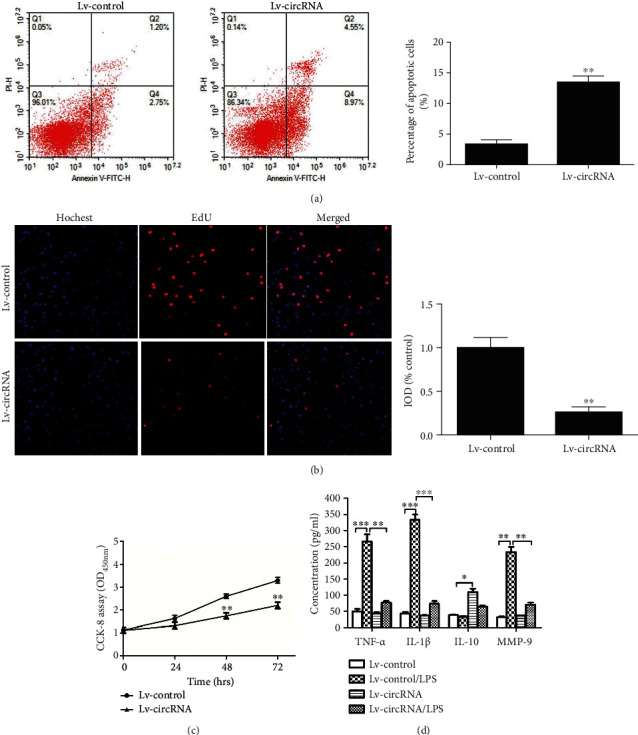
circRNA_17725 regulated macrophage proliferation, apoptosis, and inflammation. (a) Flow cytometry: Lv-circRNA_17725 plasmids promoted the apoptosis of macrophages induced by H_2_O_2_ at 4 h (^∗∗^*P* < 0.01; *n* = 3). (b) EdU: Lv-circRNA_17725 plasmids inhibited the proliferation of macrophages at 48 h (20x; ^∗∗^*P* < 0.01; *n* = 3). (c) CCK-8: Lv-circRNA_17725 plasmids inhibited the proliferation of macrophages in a time-dependent way (^∗∗^*P* < 0.01; *n* = 3). (d) ELISA: Lv-circRNA_17725 plasmids decreased TNF-*α*, IL-1*β*, and MMP-9 but increased IL-10 in cells cultural supernatant (^∗^*P* < 0.05, ^∗∗^*P* < 0.01, and ^∗∗∗^*P* < 0.001; *n* = 3).

Here, we have performed a series of experiments to elucidate the role and mechanism of circRNA_17725 on macrophage growth and functions. circRNA_17725 was overexpressed in Raw264.7 macrophages by use of lentivirus plasmids (Figure 4(a)). The test of FISH has suggested circRNA_17725 was primarily located in macrophage cytoplasm (Figure 4(b)). The apoptosis of Lv-circRNA_17725 plasmids-intervened macrophage was obviously promoted compared with that of Lv-control plasmid-treated cells, which was demonstrated by flow cytometry (Figure 5(a)). However, results of EdU and CCK-8 cell proliferation detections showed that circRNA_17725 significantly inhibited the proliferation of Raw264.7 macrophage in a time-dependent manner (Figures 5(b) and 5(c)). Moreover, inflammatory cytokines of IL-1*β*, TNF-*α*, and MMP-9 in the supernatant of Lv-circRNA_17725 plasmid-treated macrophage was significantly decreased, although they were activated by LPS, a classic stimulator for macrophage activation (Figure 5(d)). IL-10 was reversely enhanced in the cultural supernatant of Lv-circRNA_17725 plasmids treated macrophage (Figure 5(d)). Taken together, circRNA_17725 can regulate macrophage proliferation, apoptosis, and inflammatory response *in vitro*, suggesting a pivotal role of circRNA_17725 in macrophage-mediated inflammation in RA. Nonetheless, its altering effects on macrophage functional phenotypes and the potential molecular mechanism are needed to elucidate in the following experiments.

### 3.5. circRNA_17725 Promoted M2 Polarization via Targeting miR-4668-5p/FAM46C

**Figure 6:**
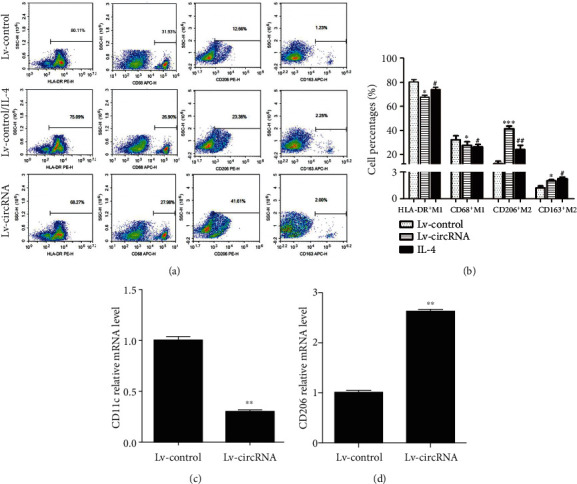
circRNA_17725 promoted macrophage polarization towards M2. (a) Flow cytometry: circRNA_17725 promoted CD206^+^M2 and CD163^+^M2 cells while inhibited HLA-DR^+^M1 and CD68^+^M1 cells polarization (representative pictures). (b) Cell percentages of CD206^+^M2, CD163^+^M2, HLA-DR^+^M1, and CD68^+^M1 cells determined by flow cytometry (vs. Lv-control: ^∗^*P* < 0.05, ^∗∗∗^*P* < 0.001; vs. Lv-control: ^#^*P* < 0.05, ^##^*P* < 0.01; *n* = 3). (c) CD11c mRNA expression was decreased in circRNA_17725-overexpressed Raw264.7 cells (^∗∗^*P* < 0.01; *n* = 3). (d) CD206 mRNA expression was increased in circRNA_17725-overexpressed macrophages (^∗∗^*P* < 0.01; *n* = 3).

**Figure 7:**
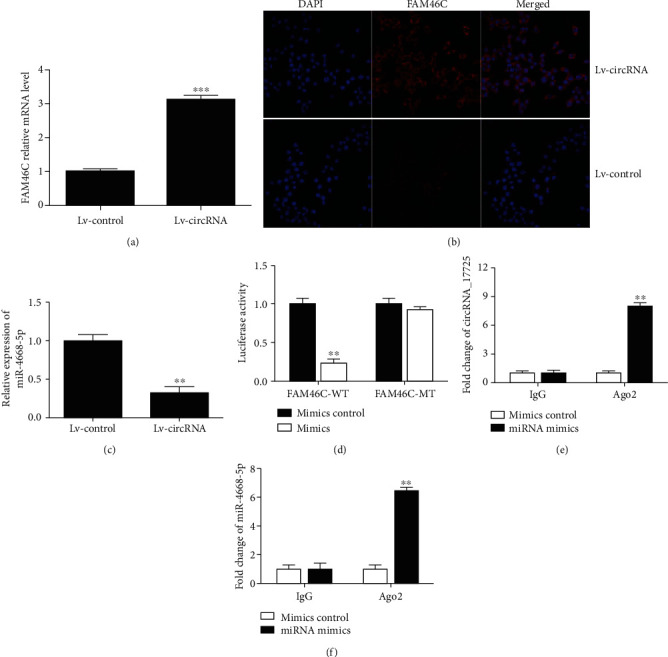
circRNA_17725 functioned as a ceRNA based on the circRNA_17725-miR-4668-5p-FAM46C network. (a) Real-time PCR: increased expression of FAM46C in Lv-circRNA-transfected Raw264.7 macrophages (^∗∗∗^*P* < 0.001; *n* = 3). (b) Cell immunofluorescence: higher expression of FAM46C in Raw264.7 macrophages transfected by Lv-circRNA plasmids (representative pictures; 20x). (c) Real-time PCR: decreased miR-4668-5p in Lv-circRNA-treated cells (^∗∗^*P* < 0.01; *n* = 3). (d) Luciferase reporter assay: miR-4668-5p mimics inhibited FAM46C expression in macrophages (^∗∗^*P* < 0.01; *n* = 3). (e) RIP test: expression of circRNA_17725 in immunoprecipitations (^∗∗^*P* < 0.01; *n* = 3). (f) RIP test: expression of miR-4668-5p in immunoprecipitations (^∗∗^*P* < 0.01; *n* = 3).

As shown in Figures 6(a) and 6(b), IL-4 could induce M2 polarization of Raw264.7 cells. Overexpression of circRNA_17725 enhanced the percentages of CD206^+^M2 and CD163^+^M2 cells, but significantly inhibited HLA-DR^+^M1 and CD68^+^M1 cell percentages (Figures 6(a) and 6(b)). Besides, the mRNA level of CD206 was elevated, while CD11c mRNA expression was obviously decreased in circRNA_17725-overexpressed macrophages (Figures 6(c) and 6(d)). As a result, circRNA_17725 promoted M2 polarization *in vitro*. Nonetheless, the underlying molecular mechanism of circRNA_17725 in regulating macrophage polarization was still not clear. We further performed the following experiments to elucidate the potential mechanism underlying macrophage differentiation and polarization. Previous bioinformatics analysis had suggested the circRNA_17725/miR-4668-5p/FAM46C ceRNA network (Figure 2). In this study, Lv-circRNA-transfected Raw264.7 macrophage had upregulated expression of FAM46C but decreased expression of miR-4668-5p compared with the Lv-control group (Figures 7(a)–7(c)). Besides, the luciferase reporter assay had implicated that miR-4668-5p significantly downregulated FAM46C at the post-transcriptional level in macrophages (Figure 7(d)). Moreover, the RIP test suggested circRNA_17725 could combine with miR-4668-5p and acting as a ceRNA in macrophages (Figures 7(e) and 7(f)). Taken together, circRNA_17725 is involved in regulating macrophage polarization by targeting FAM46C through the circRNA_17725-miR-4668-5p-FAM46C ceRNA network.

### 3.6. Overexpression of circRNA_17725 in Macrophage Alleviated Arthritis in CIA Mice by Promoting M2 Polarization

**Figure 8:**
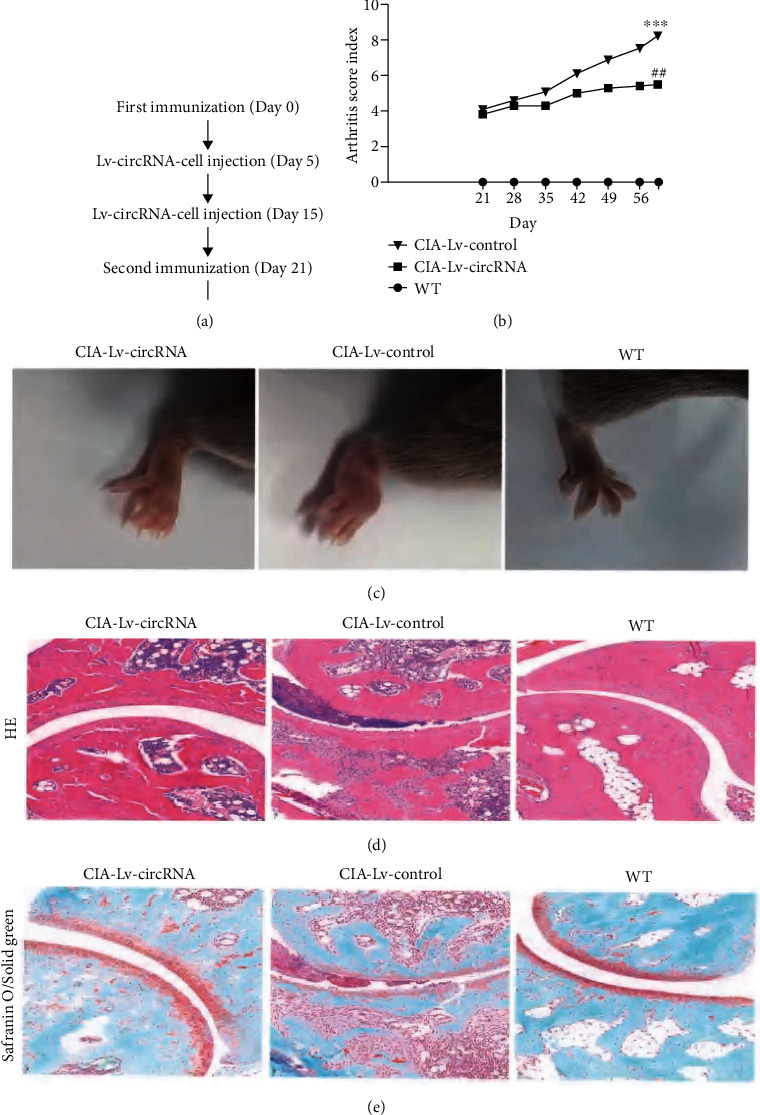
circRNA_17725-overexpressed macrophage alleviated arthritis in CIA mice. (a) CIA mouse model construction flowchart (6 mice/group). (b) Arthritis score: circRNA_17725-overexpressed macrophage-treated CIA mice had lower arthritis score (vs. the WT group, ^∗∗∗^*P* < 0.001; vs. the CIA-Lv-control group, ^##^*P* < 0.01). (c) Representative pictures: less redness, swelling, and alleviated arthritis of CIA mice treated by circRNA_17725-overexpressed macrophages. (d) Representative pictures of HE staining of joint tissues from CIA mice treated by circRNA_17725-overexpressed macrophages (20x). (e) Representative Safranin O/Solid green staining pictures: less severe cartilaginous injury and bone damages in the joint tissues of CIA mice treated by Lv-circRNA-transfected macrophages (20x).

**Figure 9:**
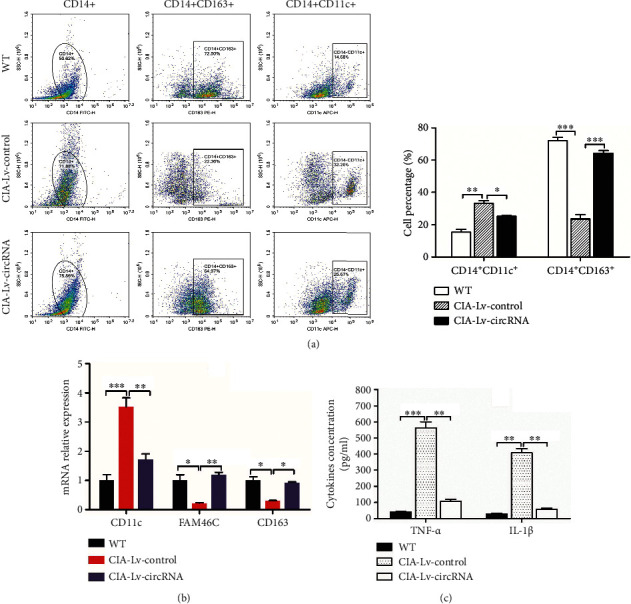
circRNA_17725 promoted M2 polarization in CIA mice. (a) Flow cytometry: CD14^+^CD163^+^ cells and CD14^+^CD11c^+^ cells infiltration in spleens from CIA mice (^∗^*P* < 0.05, ^∗∗^*P* < 0.01, and ^∗∗∗^*P* < 0.001; 6 mice/group, *n* = 3). (b) Real-time PCR: CD11c, CD163, and FAM46C mRNA expressions in spleen mononuclear cells of CIA mice (^∗^*P* < 0.05, ^∗∗^*P* < 0.01, and ^∗∗∗^*P* < 0.001; 6 mice/group, *n* = 3). (c) ELISA: decreased TNF-*α* and IL-1*β* in the plasm from Lv-circRNA-transfected macrophage-treated CIA mice (^∗∗^*P* < 0.01, ^∗∗∗^*P* < 0.001; 6 mice/group, *n* = 3).

Figure 8(a) showed the flowchart for the construction and intervention of CIA mice. As shown in Figure 8(b), circRNA_17725 decreased the mean score of arthritis and prolonged the time of arthritis first occurred in CIA mice treated by circRNA_17725-overexpressed Raw264.7 macrophages. Less redness and swelling of mouse joints were observed in CIA mice administrated by Lv-circRNA-intervened macrophages compared with those in control mice (Figure 8(c)). As demonstrated by HE staining, less subchondral bone erosions, synovitis, and inflammatory lymphocyte infiltration were found in the joint tissue slices of CIA mice intervened by circRNA_17725-overexpressed macrophages (Figure 8(d)). Besides, the Safranin O/Solid green staining had shown less severe cartilaginous injury and bone damages in the joint tissue slices of CIA mice treated by Lv-circRNA_17725-transfected macrophages compared with those in Lv-control-transfected macrophage-treated CIA mice (Figure 8(e)). Moreover, the flow cytometry analysis showed that increased CD163^+^ M2-type cells but decreased CD11c^+^ M1-type cells were infiltrated in spleens of CIA mice treated by Lv-circRNA_17725-transfected macrophages (Figure 9(a)). Similarly, the real-time PCR had suggested lower levels of CD11c mRNA but higher levels of CD163 and FAM46C mRNAs in spleen mononuclear cells of macrophage-treated CIA mice compared with untreated CIA mice, which had been treated by Lv-circRNA control plasmid-transfected macrophages through tail intravenous injection (Figure 9(b)). Moreover, reduced production of TNF-*α* and IL-1*β* was observed in the plasm samples from Lv-circRNA_17725-transfected macrophage-treated CIA mice (Figure 9(c)). Taken together, circRNA_17725 protected against synovitis, joint injuries, and bone destruction in vivo by inducing macrophage polarization towards M2 through the circRNA_17725-miR-4668-5p-FAM46C signaling axis.

## 4. Discussion

With the progress of molecular biology techniques, the role of ncRNAs in autoimmune diseases, cancers, and inflammation-associated diseases has been elucidated, some of which may serve as ideal markers for disease diagnosis and treatment. In previous studies, the specific expression profiles of ncRNAs in RA patients have been identified, primarily including specific lncRNAs and miRNAs. Among them, lncRNA HIX003209, miR-6089, and miR-548a-3p have been demonstrated to be involved in regulating inflammation and autoimmunity [13–15]. In particular, lncRNA HIX003209 can sponge with miR-6089 and function as a ceRNA in macrophage response in RA [13]. Exosomes-delivering miR-6089 and miR-548a-3p can inhibit the generation of inflammatory mediators in macrophages, which thus alleviates arthritis [14, 15]. circRNAs are ncRNAs with closed circular structure, which account for the majority of ncRNAs. They are stably expressed and not easy to degradation. circRNAs play critical roles in various pathophysiological processes. Accumulating studies have reported that some specific circRNAs are dysregulated and associated with the disease activity of RA [16, 17]. We have also found that circRNA_09505 was upregulated in RA and participated in macrophage-mediated immune and inflammatory response in RA [12]. Accordingly, identification of novel circRNAs in RA may provide insight into the pathogenesis and biological therapy of RA.

Macrophages are heterogeneous cells with different phenotypes and functions, which can be differentiated into classically activated M1 and alternatively activated M2 macrophages under the modifying effects of diverse mediators in the microenvironment. Increased levels of proinflammatory mediators from M1-type macrophages lead to inflammation and immune disorders, such as TNF-*α*, IL-6, and IL-1*β* [18]. It has been well established that M1/M2 imbalance was found in RA characterized by more M1 macrophages and fewer M2 macrophages in the immune microenvironment [19]. M1/M2 imbalance can promote Th1 and Th17 cell reactions, which thus exacerbates the immunoinflammatory response and causes synovial cell hyperplasia, synovial hypertrophy, osteoclast formation, and cartilage injury [20]. Accordingly, intervening the M1/M2 macrophage polarization balance effectively and maintaining the balance of immune microenvironment would be a promising therapeutic way for RA. It has been found that family with sequence similarity 46 member C (FAM46C), signaling, and signal transducer and activator of transcription 33 (STAT3) play important regulatory roles in regulating macrophage polarization [19, 21, 22]. Some ncRNAs have been demonstrated to regulate the macrophage phenotypic polarization and function by targeting STAT3 and other molecules associated with macrophage polarization, such as miR-221-3p and lncRNA NTT [23, 24]. Nonetheless, litter is known about the impact of circRNAs in regulating macrophage phenotypes in RA. In this study, we have found that circRNA_17725 could reduce macrophage proliferation but promote cell apoptosis. Besides, circRNA_17725 reduced the expression of inflammatory factors, such as TNF-*α* and IL-1*β* in RA. Moreover, circRNA_17725 played a protective role by promoting M2 polarization in vitro and in vivo, suggesting the crucial effect of circRNA in regulating inflammation and autoimmunity in RA.

It has been demonstrated that the expression profile of circRNAs is different between M1 macrophage and M2 macrophage [25], suggesting the diverse and complicated regulatory effects of circRNAs on macrophage phenotypes and functions. M1/M2 bias leads to chronic inflammation and metabolic imbalance in SLE, which influences the balance of immune microenvironment in targeted organs including kidneys [26]. Song et al. have reported that circCdyl played a pivotal role in abdominal aortic aneurysm (AAA) by promoting M1 macrophage inflammatory response and inducing M1 polarization [27]. circ_0000518 has been demonstrated to promote macrophage/microglia M1 polarization in multiple sclerosis [28]. A recent study has also suggested the crucial role of circ_0005567 in inhibiting chondrocyte apoptosis and the progression of osteoarthritis by promoting M2 polarization via the miR-492/SOCS2 signaling axis [29]. However, the molecular mechanism of circRNA in regulating M1/M2 polarization in RA is rarely reported. In this study, we have found that circRNA_17725 was significantly downregulated in RA. It was capable of inhibiting macrophage proliferation and inflammatory response, implicating a pivotal regulatory effect of circRNA_17725 on macrophage-mediated inflammation in RA. Moreover, it could elevate the expression of M2-associated molecule FAM46C and thus promote M2-type cell response in CIA mice by increasing CD163^+^ M2 macrophages but decreasing CD11c^+^ M1 macrophage infiltration in spleens of CIA mice. Taken together, this study has implicated the protective role of circRNA_17725 in RA and its critical effects on macrophage phenotypic plasticity.

The functions of circRNAs are different according to their localization in cells [30, 31]. Most circRNAs molecules are localized in the cytoplasm and exert effects by RNA-RNA interactions including sponging with miRNAs and antagonizing their posttranscriptional regulatory effects on the downstream mRNAs [32, 33]. Nonetheless, some circRNAs can function by directly binding to proteins through RNA-protein interactions [28, 34]. In a previous study, we found that circRNA_09505 could aggravate inflammation and joint damage by regulating macrophage-mediated immunoinflammatory response in CIA mice by acting as a ceRNA for miR-6089 via the AKT1/NF-*κ*B axis [12], which suggests the pivotal mechanism of circRNA as a ceRNA molecule in immune cells. The altering effect of circRNA in regulating macrophage phenotypic polarization warrants in depth investigation. In this study, the findings have strongly supported that overexpression of circRNA_17725 could elevate CD206^+^M2 and CD163^+^M2 cells percentages, while significantly inhibit HLA-DR^+^M1 and CD68^+^M1 cells percentages. Besides, miR-4668-5p could downregulate the expression of FAM46C in macrophage, while circRNA_17725 served as a ceRNA by sponging miR-4668-5p and promoted FAM46C expression in macrophages. All the findings have shed some light on the pathogenesis of RA. It also provides novel ideas for investigating more promising immunotherapy for RA by targeting certain checkpoint molecules in this axis. Nevertheless, there are some drawbacks in this study. On the one hand, the role of circRNA_17725 in regulating macrophage polarization should be further evaluated in the local immune balance of joints. On the other hand, future studies are recommended to investigate the modifying effects of circRNA_17725 on influencing cartilage damages and articular repair. Last but not the least, the downstream signaling pathway and key checkpoints of circRNA_17725-miR-4668-5p-FAM46C axis warrant to be elucidated in the future.

In conclusion, this study has implicated that circRNA_17725 protected against synovitis, joint injuries and bone destruction by inducing macrophage polarization towards M2 through the circRNA_17725-miR-4668-5p-FAM46C signaling axis in RA. circRNA_17725 might be serve as a promising target for the biotherapy of RA.

## Data Availability

All data and materials in this study have been included in this paper. Further inquiries can be directed to the corresponding authors.

## Abbreviations

circRNAs: Circular RNAs
RA: Rheumatoid arthritis
RIP: RNA binding protein immunoprecipitation
CIA: Collagen-induced arthritis
ncRNAs: Noncoding RNAs
circRNA: Circular RNA
lncRNA: Long chain noncoding RNAs
miRNA: MicroRNAs
PBMCs: Peripheral blood mononuclear cells
ceRNA: Competitive endogenous RNA
SLE: Systemic lupus erythematosus
MS: Multiple sclerosis
FISH: RNA fluorescence in situ hybridization
FAM46C: Sequence similarity 46 member C
STAT3: Signaling and signal transducer and activator of transcription 3
AAA: Abdominal aortic aneurysm.
